# The Gut Bacterial Community Potentiates Clostridioides difficile Infection Severity

**DOI:** 10.1128/mbio.01183-22

**Published:** 2022-07-20

**Authors:** Nicholas A. Lesniak, Alyxandria M. Schubert, Kaitlin J. Flynn, Jhansi L. Leslie, Hamide Sinani, Ingrid L. Bergin, Vincent B. Young, Patrick D. Schloss

**Affiliations:** a Department of Microbiology and Immunology, University of Michigan, Ann Arbor, Michigan, USA; b Division of Infectious Diseases, Department of Internal Medicine, University of Michigan Medical School, Ann Arbor, Michigan, USA; c Unit for Laboratory Animal Medicine, University of Michigan, Ann Arbor, Michigan, USA; Rutgers University

**Keywords:** CDI, *Clostridium difficile*, human microbiome, humanized mice, microbial ecology

## Abstract

The severity of Clostridioides difficile infections (CDI) has increased over the last few decades. Patient age, white blood cell count, and creatinine levels as well as C. difficile ribotype and toxin genes have been associated with disease severity. However, it is unclear whether specific members of the gut microbiota are associated with variations in disease severity. The gut microbiota is known to interact with C. difficile during infection. Perturbations to the gut microbiota are necessary for C. difficile to colonize the gut. The gut microbiota can inhibit C. difficile colonization through bile acid metabolism, nutrient consumption, and bacteriocin production. Here, we sought to demonstrate that members of the gut bacterial communities can also contribute to disease severity. We derived diverse gut communities by colonizing germfree mice with different human fecal communities. The mice were then infected with a single C. difficile ribotype 027 clinical isolate, which resulted in moribundity and histopathologic differences. The variation in severity was associated with the human fecal community that the mice received. Generally, bacterial populations with pathogenic potential, such as *Enterococcus*, *Helicobacter*, and Klebsiella, were associated with more-severe outcomes. Bacterial groups associated with fiber degradation and bile acid metabolism, such as *Anaerotignum*, *Blautia*, *Lactonifactor*, and *Monoglobus*, were associated with less-severe outcomes. These data indicate that, in addition to the host and C. difficile subtype, populations of gut bacteria can influence CDI disease severity.

## INTRODUCTION

Clostridioides difficile infections (CDI) have increased in incidence and severity since C. difficile was first identified as the cause of antibiotic-associated pseudomembranous colitis (1). CDI disease severity can range from mild diarrhea to toxic megacolon and death. The Infectious Diseases Society of America (IDSA) and Society for Healthcare Epidemiology of America (SHEA) guidelines define severe CDI in terms of a white blood cell count greater than 15,000 cells/mm^3^ and/or a serum creatinine level of greater than 1.5 mg/dL. Patients who develop shock or hypotension, ileus, or toxic megacolon are considered to have fulminant CDI (2). Since these measures are CDI outcomes, they have a limited ability to predict risk of severe CDI when the infection is first detected. Schemes have been developed to score a patient’s risk for severe CDI outcomes based on clinical factors but have not been robust for broad application (3). Thus, we have limited ability to prevent patients from developing severe CDI.

Missing from CDI severity prediction models are the effects of the indigenous gut bacteria. C. difficile interacts with the gut community in many ways. The indigenous bacteria of a healthy intestinal community prevent C. difficile from infecting the gut (4). A range of mechanisms can disrupt this inhibition, including antibiotics, medications, and dietary changes, and lead to increased susceptibility to CDI (5–7). Once C. difficile overcomes the inhibition and colonizes the intestine, the indigenous bacteria can either promote or inhibit C. difficile by producing molecules or modifying the environment (8, 9). Bile acids metabolized by the gut bacteria can inhibit C. difficile growth and affect toxin production (4, 10, 11). Bacteria in the gut also can compete more directly with C. difficile through antibiotic production or nutrient consumption (12–14). While the relationship between the gut bacteria and C. difficile has been established, the effect the gut bacteria can have on CDI disease severity is unclear.

Recent studies have demonstrated that when mice with diverse microbial communities were challenged with a high-toxigenicity strain, it resulted in varied disease severity (15), and when challenged with a low-toxigenicity strain, members of the gut microbial community were associated with variation in colonization (16). Here, we sought to further elucidate the relationship between members of the gut bacterial community and CDI disease severity when challenged with a highly toxigenic strain, C. difficile ribotype 027 (RT027). We hypothesized that since specific groups of gut bacteria affect the metabolism of C. difficile and its clearance rate, specific groups of bacteria are associated with variation in CDI disease severity. To test this hypothesis, we colonized germfree C57BL/6 mice with human fecal samples to create varied gut communities. We then challenged the mice with C. difficile RT027 and followed the mice for the development of severe outcomes of moribundity and histopathologic cecal tissue damage. Since the murine host and C. difficile isolate were the same and only the gut community varied, the variation in disease severity we observed was attributable to the gut microbiome.

## RESULTS

### C. difficile is able to infect germfree mice colonized with human fecal microbial communities without antibiotics.

**FIG 1:**
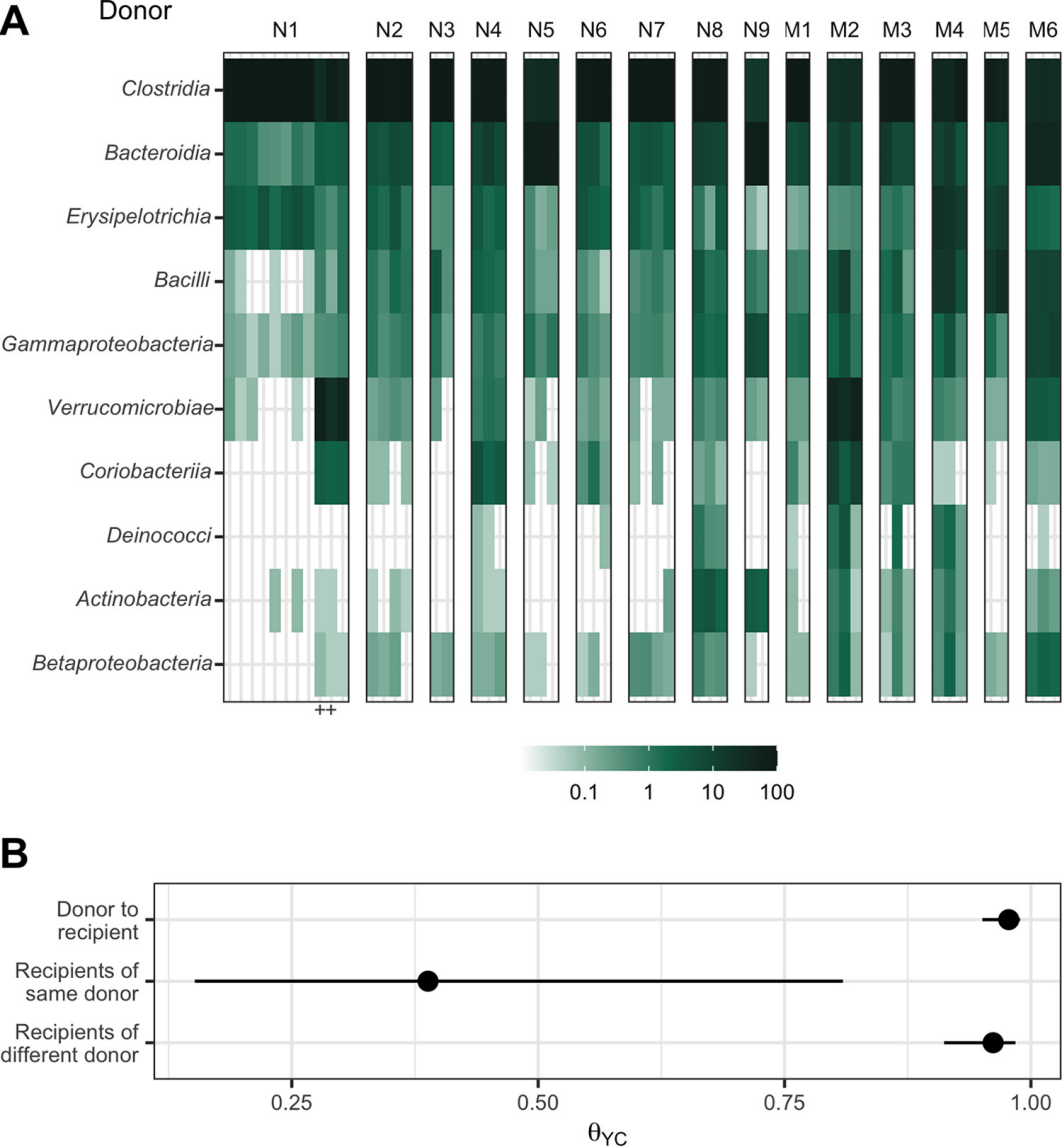
Human fecal microbial communities established diverse gut bacterial communities in germfree mice. (A) Relative abundances of the 10 most abundant bacterial classes observed in the feces of previously germfree C57BL/6 mice 14 days postcolonization with human fecal samples (i.e., day 0 relative to C. difficile challenge). Each column of abundances represents an individual mouse. Mice that received the same donor feces are grouped together and labeled above with a letter (“N” for nonmoribund mice and “M” for moribund mice) and number (ordered by mean histopathologic score of the donor group). “+” indicates the mice that did not have detectable C. difficile CFU (Fig. 2). (B) Medians (points) and interquartile ranges (lines) of β diversity (θ_YC_) between an individual mouse and either all others that were inoculated with feces from the same donor or inoculated with feces from a different donor. The β diversity among the same-donor comparison group was significantly less than the β diversity of either the different-donor group or the overall donor community (*P* < 0.05, calculated by Wilcoxon rank sum test).

To produce gut microbiomes with greater variation than those found in conventional mouse colonies, we colonized germfree mice with bacteria from human feces (17). We inoculated germfree C57BL/6 mice with homogenized feces from each of 15 human fecal samples via oral gavage. These human fecal samples were selected because they represented diverse community structures based on community clustering (18). After the gut communities had colonized for 2 weeks, we confirmed them to be C. difficile negative by culture (19). We then surveyed the bacterial members of the gut communities by 16S rRNA gene sequencing of murine fecal pellets (Fig. 1A). The bacterial communities from each mouse grouped more closely to those communities from mice that received the same human fecal donor community than to the mice who received a different human fecal donor community (Fig. 1B). The communities were primarily composed of populations of *Clostridia*, *Bacteroidia*, *Erysipelotrichia*, *Bacilli*, and *Gammaproteobacteria*. However, the gut bacterial communities of each donor group of mice harbored unique relative abundance distributions of the shared bacterial classes.

**FIG 2:**
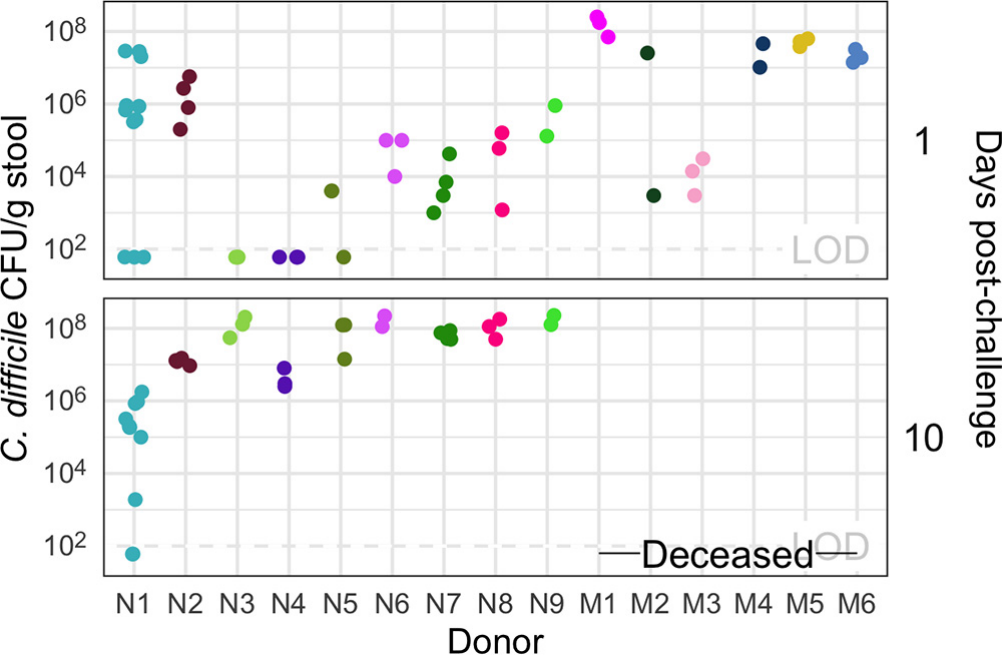
All donor groups resulted in C. difficile infection, but with different outcomes. The number of C. difficile CFU per gram of stool was measured the day after challenge with 10^3^ C. difficile RT027 clinical isolate 431 spores and at the end of the experiment, 10 days postchallenge. Each point represents an individual mouse. Mice are grouped by donor and labeled by the donor letter (“N” for nonmoribund mice and “M” for moribund mice) and number (ordered by mean histopathologic score of the donor group). Points are colored by donor group. Mice from donor groups N1 through N6 succumbed to the infection prior to day 10 and were not plated on day 10 postchallenge. LOD, limit of detection. “—Deceased—” indicates mice were deceased at that time point, so no sample was available.

Next, we tested this set of mice with their human-derived gut microbial communities for susceptibility to C. difficile infection. A typical mouse model of CDI requires pretreatment of conventional mice with antibiotics, such as clindamycin, to become susceptible to C. difficile colonization (20, 21). However, we wanted to avoid modifying the gut communities with an antibiotic to maintain their unique microbial compositions and ecological relationships. Since some of these communities came from people at increased risk of CDI, such as recent hospitalization or antibiotic use (18), we tested whether C. difficile was able to infect these mice without an antibiotic perturbation. We hypothesized that C. difficile would be able to colonize the mice who received their gut communities from a donor with a perturbed community. Mice were challenged with 10^3^ C. difficile RT027 clinical isolate spores. The mice were followed for 10 days postchallenge, and their stool was collected and plated for C. difficile CFU to determine the extent of the infection. Surprisingly, communities from all donors were able to be colonized (Fig. 2). Two mice were able to resist C. difficile colonization: both received their community from donor N1 (“N” represents nonmoribund), which may be attributed to experimental variation since this group also had more mice. By colonizing germfree mice with different human fecal communities, we were able to generate diverse gut communities in mice, which were susceptible to C. difficile infection without further modification of the gut community.

### Infection severity varies by initial community.

**FIG 3:**
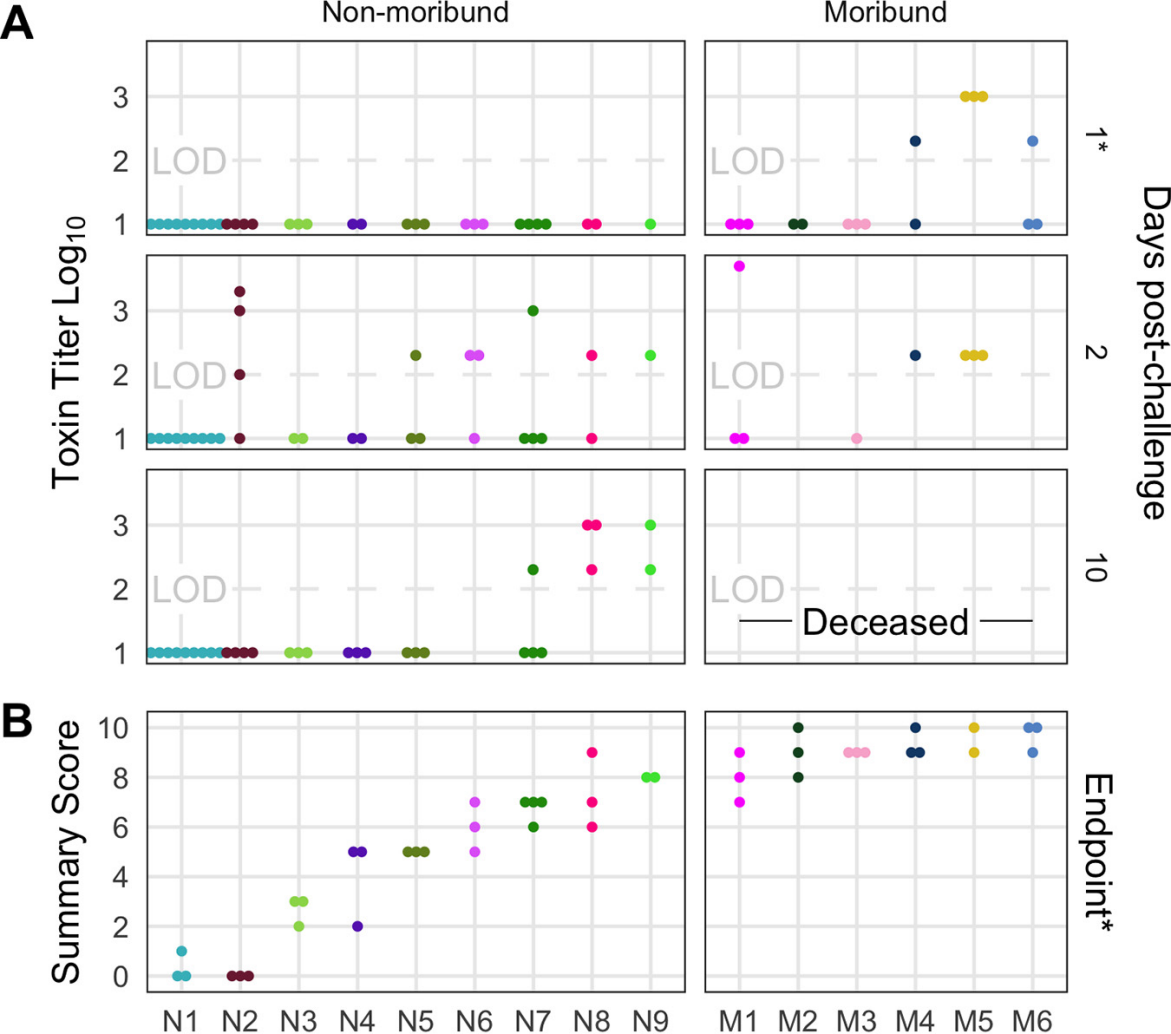
Histopathologic scores and toxin activities varied across donor groups. (A) Fecal toxin activity was detected in some mice post-C. difficile challenge in both moribund and nonmoribund mice. (B) Cecum scored for histopathologic damage from mice at the end of the experiment. Samples were collected for histopathologic scoring on day 10 postchallenge for nonmoribund mice or the day the mouse succumbed to the infection for the moribund group (day 2 or 3 postchallenge). Each point represents an individual mouse. Mice are grouped by donor and labeled by the donor letter (“N” for nonmoribund mice and “M” for moribund mice) and number (ordered by mean histopathologic score of the donor group). Points are colored by donor group. Mice in group N1 that have a summary score of 0 are the mice that did not have detectable C. difficile CFU (Fig. 2). Missing points are from mice that had insufficient fecal sample collected for assaying toxin or cecum for histopathologic scoring. *, significant difference between nonmoribund and moribund groups of mice by Wilcoxon test (*P* < 0.002).

After we challenged the mice with C. difficile, we investigated the outcome from the infection and its relationship to the initial community. We followed the mice for 10 days postchallenge for colonization density, toxin production, and mortality. Seven mice, with communities from donors N1, N3, N4, and N5 were not colonized at detectable levels on the day after C. difficile challenge but were infected (>10^6^) by the end of the experiment. All mice that received their community from donors M1 through M6 (“M” represents moribund) succumbed to the infection and became moribund within 3 days postchallenge. The remaining mice, except the uninfected donor N1 mice, maintained C. difficile infection through the end of the experiment (Fig. 2). At 10 days postchallenge, or earlier for the moribund mice, mice were euthanized, fecal material was assayed for toxin activity, and cecal tissue was collected and scored for histopathologic signs of disease (Fig. 3). Overall, there was greater toxin activity detected in the stool of the moribund mice (see Fig. S1 in the supplemental material). However, when looking at each group of mice, we observed a range of toxin activities for both the moribund and nonmoribund mice (Fig. 3A). Nonmoribund mice with communities from donors N2 and N5 through N9 had comparable toxin activity to the moribund mice at 2 days postchallenge. Additionally, not all moribund mice had toxin activity detected in their stool. Next, we examined the cecal tissue for histopathologic damage. Moribund mice had high levels of epithelial damage, tissue edema, and inflammation (Fig. S2), similar to previously reported histopathologic findings for C. difficile RT027 (22). As observed with toxin activity, the moribund mice had higher histopathologic scores than the nonmoribund mice (*P* < 0.001). However, unlike the toxin activity, all moribund mice had consistently high histopathologic summary scores (Fig. 3B). The nonmoribund mice (representing donor groups N1 through N9) had a range of tissue damage from none detected to levels similar to those in the moribund mice, which grouped by community donor. Together, the toxin activity, histopathologic score, and moribundity showed variation across the donor groups but were largely consistent within each donor group.

10.1128/mbio.01183-22.1 FIG S1 Toxin detection in mice based on outcome of the infection. Comparison of the distribution of numbers of either nonmoribund or moribund mice in which toxin was detected in the first 3 days postinfection. Bars are colored by whether toxin was detected in stool from the mouse (dark purple) or not (light purple). Moribund mice had significantly more mice with toxin detected (*P* < 0.008) by Pearson’s chi-square test. Download FIG S1, TIF file, 0.1 MB. Copyright © 2022 Lesniak et al. 2022 Lesniak et al. https://creativecommons.org/licenses/by/4.0/ This content is distributed under the terms of the Creative Commons Attribution 4.0 International license.

10.1128/mbio.01183-22.2 FIG S2 Histopathologic score of tissue damage at the endpoint of the infection. Tissue collected at the endpoint, either day 10 postchallenge (nonmoribund) or on the day mice succumbed to infection (moribund), were scored from histopathologic damage. Each point represents an individual mouse. Mice (points) are grouped and colored by their human fecal community donor. Missing points are from mice that had insufficient sample for histopathologic scoring. *, significant difference between nonmoribund and moribund groups of mice by Wilcoxon test (*P* < 0.002). Download FIG S2, TIF file, 0.3 MB. Copyright © 2022 Lesniak et al. 2022 Lesniak et al. https://creativecommons.org/licenses/by/4.0/ This content is distributed under the terms of the Creative Commons Attribution 4.0 International license.

### Microbial community members explain variation in CDI severity.

**FIG 4:**
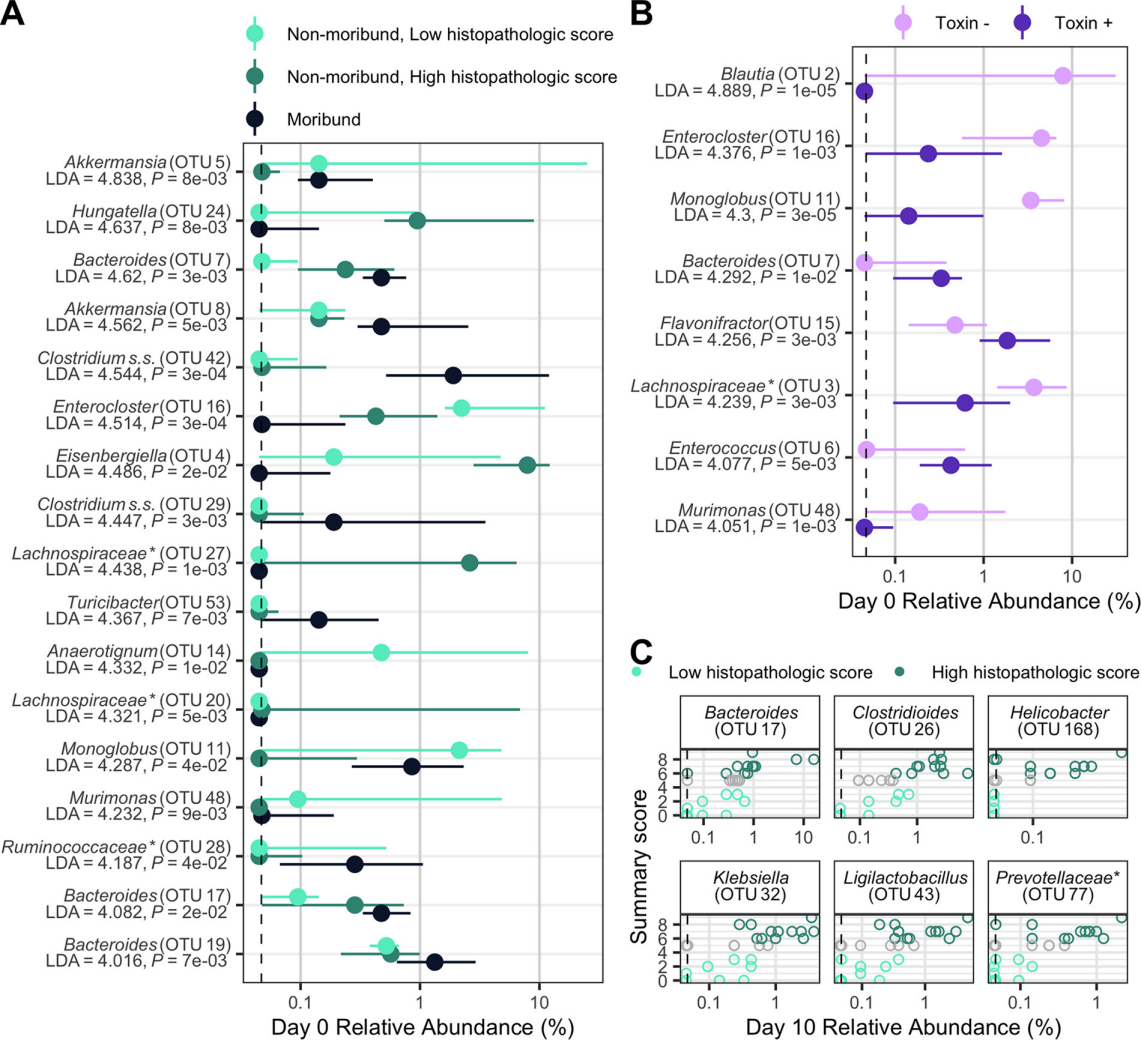
Individual fecal bacterial community members of the murine gut associated with C. difficile infection outcomes. (A and B) Relative abundance of OTUs at the time of C. difficile challenge (day 0) that varied significantly by the moribundity and histopathologic summary score or with detected toxin by LEfSe analysis. The median (points) and interquartile range (lines) are plotted. (A) Day 0 relative abundances were compared across infection outcome of moribund (colored black) or nonmoribund with either a high histopathologic score (score greater than the median score of 5, colored green) or a low histopathologic summary score (score less than the median score of 5, colored light green). (B) Day 0 relative abundances were compared between mice in which toxin activity was detected (Toxin +, colored dark purple) and which no toxin activity was detected (Toxin −, colored light purple). (C) Day 10 bacterial OTU relative abundances correlated with histopathologic summary score. Data for each mouse are plotted and colored according to their categorization in panel A. Points at the median score of 5 (gray points) were not included in panel A. Spearman’s correlations were statistically significant after Benjamini-Hochberg correction for multiple comparisons. All bacterial groups are ordered by the LDA score. *, the bacterial group was unclassified at lower taxonomic classification ranks.

We next interrogated the bacterial communities at the time of C. difficile challenge (day 0) for their relationship to infection outcomes using linear discriminant analysis (LDA) effect size (LEfSe) analysis to identify individual bacterial populations that could explain the variation in disease severity. We split the mice into groups by severity level based on moribundity or 10-day postinfection (dpi) histopathologic score for nonmoribund mice. This analysis revealed bacterial operational taxonomic units (OTUs) that were significantly different at the time of challenge by the disease severity (Fig. 4A). OTUs associated with *Akkermansia*, *Bacteroides*, *Clostridium sensu stricto*, and *Turicibacter* were detected at higher relative abundances in the mice that became moribund. OTUs associated with *Anaerotignum*, *Enterocloster*, and *Murimonas* were more abundant in the nonmoribund mice that would develop low intestinal injury. To understand the role of toxin activity in disease severity, we applied LEfSe to identify the OTUs at the time of challenge that most likely explain the differences between communities that had toxin activity detected at any time point and those that did not (Fig. 4B). An OTU associated with *Bacteroides*, OTU 7, which was associated with the presence of toxin, was also associated with moribundity. Likewise, OTUs associated with *Enterocloster* and *Murimonas* that were associated with no detected toxin also exhibited greater relative abundance in communities from nonmoribund mice with a low histopathologic score. We tested for correlations between the endpoint (10 dpi) relative abundances of OTUs and the histopathologic summary score (Fig. 4C). The endpoint relative abundance of *Bacteroides* OTU 17 was positively correlated with histopathologic score, as its day 0 relative abundance did with disease severity (Fig. 4A). The population of OTU 17 was also increased in the group of mice with detectable toxin. We also tested for correlations between the endpoint relative abundances of OTUs and toxin activity, but none were significant. Finally, we tested for associations between temporal changes and disease severity (Fig. S4). Most groups of bacteria maintained higher relative abundance than the other outcome groups from day 0 through the end of the experiment. This analysis identified bacterial populations that were associated with the variation in moribundity, histopathologic score, and toxin.

10.1128/mbio.01183-22.4 FIG S4 Temporal dynamics of OTUs that differed between histopathologic summary scores. Relative abundance of OTUs on each day relative to the time of C. difficile challenge (day 0) that have a significantly different temporal trend by the histopathologic summary score by LEfSe analysis. Medians (points) and interquartile ranges (lines) of relative abundances are plotted. Points and lines are colored by infection outcome of moribund (colored black) or nonmoribund with either a high histopathologic score (score greater than the median score of 5, colored green) or a low histopathologic summary score (score less than the median score of 5, colored light green). Download FIG S4, TIF file, 2.0 MB. Copyright © 2022 Lesniak et al. 2022 Lesniak et al. https://creativecommons.org/licenses/by/4.0/ This content is distributed under the terms of the Creative Commons Attribution 4.0 International license.

**FIG 5:**
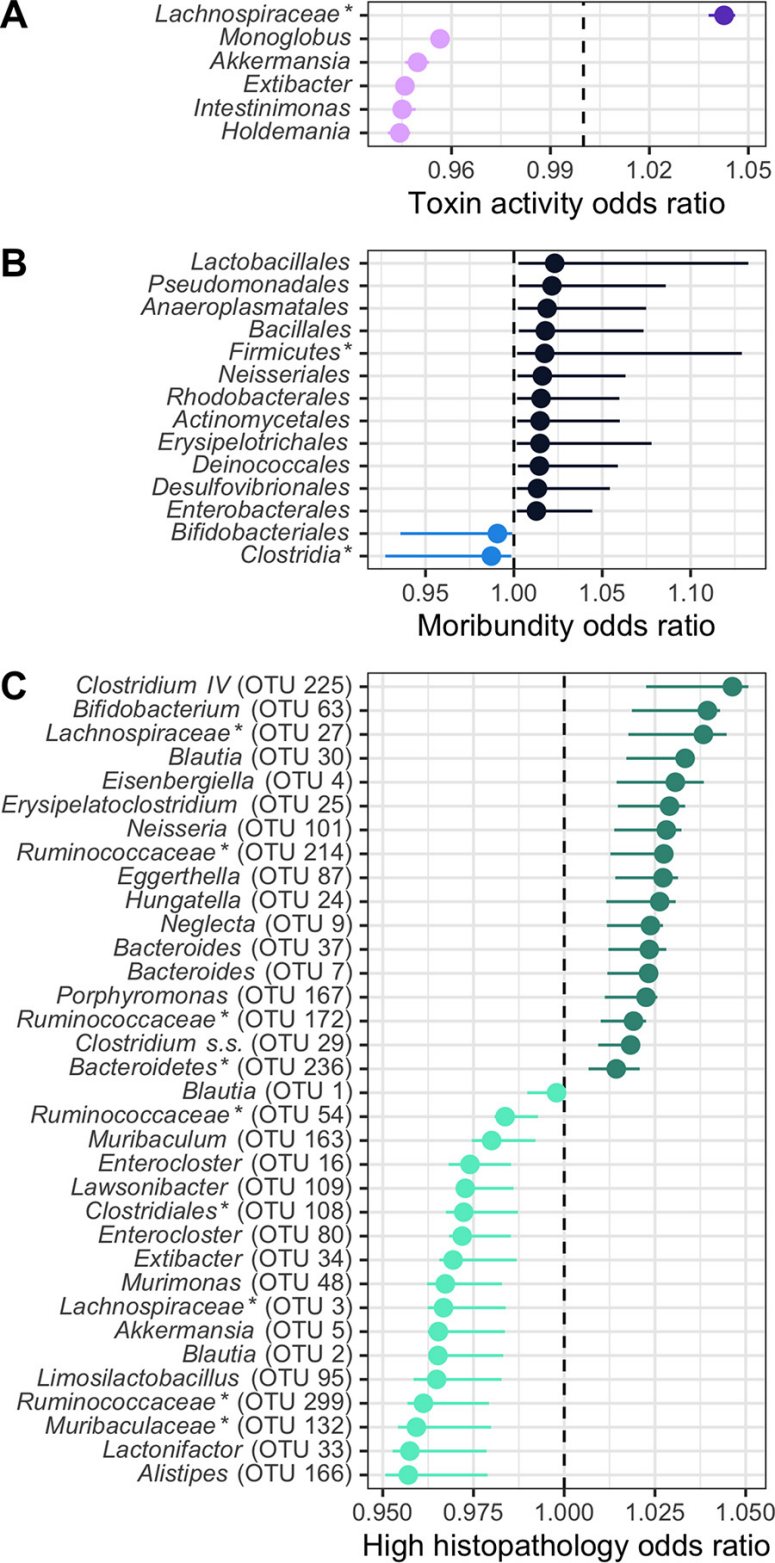
Fecal bacterial community members of the murine gut at the time of C. difficile infection predicted outcomes of the infection. On the day of infection (day 0), bacterial community members grouped by different classification rank were modeled with logistic regression to predict the infection outcome. The models used the highest taxonomic classification rank without a decrease in performance. Models used all community members, but plotted are those members with a mean odds ratio not equal to 1. The medians (solid points) and interquartile ranges (lines) of the odds ratio are plotted. Bacterial groups are ordered by their odds ratio. *, bacterial group was unclassified at lower taxonomic classification ranks. (A) Bacterial members grouped by genus predicted which mice would have toxin activity detected at any point throughout the infection. Data with a decreased probability of toxin activity are colored light purple, and those with an increased probability of toxin activity are colored dark purple. (B) Bacterial members grouped by order predicted which mice would become moribund. Data with a decreased probability of moribundity are colored light blue, and those with an increased probability of moribundity are colored dark blue. (C) Bacterial members grouped by OTU predicted if the mice would have a high (greater than the median score of 5) or low (less than the median score of 5) histopathologic summary score. Data with a decreased probability of high histopathologic score are colored light green, and those with an increased probability of high histopathologic score are colored dark green.

We next determined whether, collectively, bacterial community membership and relative abundance could be predictive of the CDI disease outcome. We trained logistic regression models with bacterial community relative abundance data from the day of colonization at each taxonomic rank to predict toxin, moribundity, and histopathologic summary score. We used the highest taxonomic classification rank that performed similar to lower ranks, which suggested the effect is associated with general attributes of the bacterial group, as opposed to specific functions of more refined grouping. For prediction of whether detectable toxin would be produced, microbial populations aggregated by genus rank classification performed similarly to models using lower taxonomic ranks (mean area under the receiver operating characteristic curve [AUROC] = 0.787 [Fig. S3]). C. difficile increased odds of producing detectable toxin when the community infected had less-abundant populations of *Monoglobus*, *Akkermansia*, *Extibacter*, *Intestinimonas*, and *Holdemania* and had more abundant populations of *Lachnospiraceae* (Fig. 5A). Next, we assessed the ability of the community to predict moribundity. Grouping of bacteria by order rank classification was sufficient to predict which mice would succumb to the infection before the end of the experiment (mean AUROC = 0.9205 [Fig. S3]). Many populations contributed to increased odds of moribundity (Fig. 5B). Populations related to *Bifidobacteriales* and *Clostridia* decreased the odds of a moribund outcome. Finally, the relative abundances of OTUs were able to predict a high or low histopathologic score 10 dpi (with histopathologic scores dichotomized as in previous analysis; mean AUROC = 0.99 [Fig. S3]). The model identified some similar OTUs to the LEfSe analysis, such as *Murimonas* (OTU 48), *Bacteroides* (OTU 7), and *Hungatella* (OTU 24). These models have shown that the relative abundances of bacterial populations and their relationships with each other could be used to predict the variation in moribundity, histopathologic score, and detectable toxin of CDI.

10.1128/mbio.01183-22.3 FIG S3 Logistic regression models predicted outcomes of the C. difficile challenge. (A to C) Taxonomic classification rank model performance. Relative abundances at the time of C. difficile challenge (day 0) of the bacterial community members grouped by different classification ranks were modeled with random forest to predict the infection outcome. The models that used the highest taxonomic classification rank performed as well as those with the lower ranks. The black rectangle highlights the classification rank used to model each outcome. For all plots, medians (large solid points), interquartile ranges (lines), and individual models (small transparent points) are plotted. (A) Toxin production modeled which mice would have toxin detected during the experiment. (B) Moribundity modeled which mice would succumb to the infection prior to day 10 postchallenge. (C) Histopathologic score modeled which mice would have a high (score greater than the median score of 5) or low (score less than the median score of 5) histopathologic summary score. Download FIG S3, TIF file, 0.6 MB. Copyright © 2022 Lesniak et al. 2022 Lesniak et al. https://creativecommons.org/licenses/by/4.0/ This content is distributed under the terms of the Creative Commons Attribution 4.0 International license.

## DISCUSSION

Challenging mice colonized with different human fecal communities with C. difficile RT027 demonstrated that variation in members of the gut microbiome affects C. difficile infection disease severity. Our analysis revealed an association between the relative abundance of bacterial community members and disease severity. Previous studies investigating the severity of CDI disease involving the microbiome have had a limited ability to interrogate this relationship between the microbiome and disease severity. Studies that have used clinical data have a limited ability to control variation in the host, microbiome, or C. difficile ribotype (23). Murine experiments typically use a single mouse colony and different C. difficile ribotypes to create differences in severity (24). Recently, our group has begun uncovering the effect microbiome variation has on C. difficile infection. We showed the variation in the bacterial communities between mice from different mouse colonies resulted in different clearance rates of C. difficile (16). We also showed varied abilities of mice to spontaneously eliminate C. difficile infection when they were treated with different antibiotics prior to C. difficile challenge (25). Overall, the results presented here have demonstrated that the gut bacterial community contributed to the severity of C. difficile infection.

C. difficile can lead to asymptomatic colonization or infections with severity ranging from mild diarrhea to death. Physicians use classification tools to identify patients most at risk of developing a severe infection using white blood cell counts, serum albumin level, or serum creatinine level (2, 26, 27). Those levels are driven by the activities in the intestine (28). Research into the drivers of this variation have revealed factors that make C. difficile more virulent. Strains are categorized for their virulence by the presence and production of the toxins TcdA, TcdB, and binary toxin and the strains’ prevalence in outbreaks, such as ribotypes 027 and 078 (20, 29–32). However, other studies have shown that disease is not necessarily linked with toxin production (33) or the strain (34). Furthermore, there is variation in the genome, growth rate, sporulation, germination, and toxin production in different isolates of a strain (35–38). This variation may help explain why severe CDI prediction tools often miss identifying many patients with CDI that will develop severe disease (3, 24, 39, 40). Therefore, it is necessary to gain a full understanding of all factors contributing to disease variation to improve our ability to predict severity.

The state of the gut bacterial community determines the ability of C. difficile to colonize and persist in the intestine. C. difficile is unable to colonize an unperturbed healthy murine gut community and is only able to become established after a perturbation (21). Once colonized, the different communities lead to different metabolic responses and dynamics of the C. difficile population (9, 25, 41). Gut bacteria metabolize primary bile acids into secondary bile acids (4, 42, 43). The concentration of these bile acids affects germination, growth, toxin production, and biofilm formation (10, 11, 44, 45). Members of the bacterial community also affect other metabolites C. difficile utilizes. Bacteroides thetaiotaomicron produces sialidases, which release sialic acid from the mucosa for C. difficile to utilize (46, 47). The nutrient environment affects toxin production (48). Thus, many of the actions of the gut bacteria modulate C. difficile in ways that could affect the infection and resultant disease.

Myriad studies have explored the relationship between the microbiome and CDI disease. Studies examining difference in disease often use different C. difficile strains or ribotypes in mice with similar microbiota as a proxy for variation in disease, such as strain 630 for nonsevere disease and RT027 for severe disease (20, 29, 30, 49). Studies have also demonstrated variation in infection through tapering antibiotic dosage (21, 25, 50) or by reducing the amount of C. difficile cells or spores used for the challenge (20, 50). These studies often either lack variation in the initial microbiome or have variation in the C. difficile infection itself, confounding any association between variation in severity and the microbiome. Recent studies have shown variation in the initial microbiome via different murine colonies or colonizing germfree mice with human feces followed by challenge with C. difficile, which resulted in varied outcomes of the infection (15, 16, 51).

Our data have demonstrated gut bacterial relative abundances are associated with variation in toxin production, histopathologic scoring of the cecal tissue, and mortality. This analysis revealed populations of *Akkermansia*, *Anaerotignum*, *Blautia*, *Enterocloster*, *Lactonifactor*, and *Monoglobus* were more abundant in the microbiome of nonmoribund mice that had low histopathologic scores and no detected toxin. The protective roles of these bacteria are supported by previous studies. *Blautia*, *Lactonifactor*, and *Monoglobus* have been shown to be involved in dietary fiber fermentation and associated with healthy communities (52–54). *Anaerotignum*, which produces short-chain fatty acids, has been associated with healthy communities (55, 56). *Akkermansia* and *Enterocloster* were also identified as more abundant in mice that had low histopathologic scores, but have contradictory supporting evidence in the current literature. In our data, a population of *Akkermansia*, OTU 5, was most abundant in the nonmoribund mice with low histopathologic scores, but moribund mice had an increased population of *Akkermansia* OTU 8. This difference could indicate either a more protective mucus layer was present, inhibiting colonization (57, 58), or mucus consumption by *Akkermansia* could have been cross-feeding C. difficile or exposing a niche for C. difficile (59–61). Similarly, *Enterocloster* was more abundant and associated with low histopathologic scores. *Enterocloster* has been associated with healthy populations and has been used to monocolonize germfree mice to reduce the ability of C. difficile to colonize (62, 63). However, *Enterocloster* has also been involved in infections such as bacteremia (64, 65). These data have exemplified populations of bacteria that have the potential to be either protective or harmful. Thus, the disease outcome is not likely based on the abundance of individual populations of bacteria; rather, it is the result of the interactions of the community.

The groups of bacteria that were associated with either a higher histopathologic score or moribundity are members of the indigenous gut community that also have been associated with disease, often referred to as opportunistic pathogens. Some of the populations of *Bacteroides*, *Enterococcus*, and Klebsiella that are associated with worse outcomes have been shown to have pathogenic potential, expand after antibiotic use, and are commonly detected in CDI cases (66–69). In addition to these populations, *Eggerthella*, *Prevotellaceae*, and *Helicobacter*, which are associated with worse outcomes, have also been associated with intestinal inflammation (70–72). Recently, Helicobacter hepaticus was shown to be sufficient to cause susceptibility to CDI in interleukin-10 (IL-10)-deficient C57BL/6 mice (73). In our experiments, when *Helicobacter* was present, the infection was more likely to result in a high histopathologic score (Fig. 4C; see Fig. S4 in the supplemental material). While we did not use IL-10-deficient mice, it is possible the bacterial community and host response are similarly modified by *Helicobacter*, allowing C. difficile infection and host damage. Aside from *Helicobacter*, these groups of bacteria that were associated with more severe outcomes did not have a conserved association between their relative abundance and the disease severity across all mice.

Since we observed groups of bacteria that were associated with less-severe disease, it may be appropriate to apply the damage response framework for microbial pathogenesis to CDI (74, 75). This framework posits that disease is not driven by a single entity—rather it is an emergent property of the responses of the host immune system, infecting microbe, C. difficile, and the indigenous microbes at the site of infection. In this set of experiments, we used the same host background, C57BL/6 mice, and the same infecting microbe, C. difficile RT027 clinical isolate 431, with different gut bacterial communities. The bacterial groups in those communities were often present in both moribund and nonmoribund mice and across the range of histopathologic scores. Thus, it was not merely the presence of the bacteria but their activity in response to the other microbes and host that affected the extent of the host damage. Additionally, while each mouse and C. difficile population had the same genetic background, they too were reacting to the specific microbial community. Different gut microbial communities can also have different effects on the host immune responses (76). Disease severity is driven by the cumulative effect of the host immune response and the activity of C. difficile and the gut bacteria. C. difficile drives host damage through the production of toxin. The gut microbiota can modulate host damage through the balance of metabolic and competitive interactions with C. difficile, such as bacteriocin production or mucin degradation, and interactions with the host, such as host mucus glycosylation or intestinal IL-33 expression (15, 77). For example, low levels of mucin degradation can provide nutrients to other community members, producing a diverse nondamaging community (78). However, if mucin degradation becomes too great, it reduces the protective function of the mucin layer and exposes the epithelial cells. This overharvesting can contribute to the host damage due to other members producing toxin. Thus, the resultant intestinal damage is the balance of all activities in the gut environment. Host damage is the emergent property of numerous damage response curves, such as one for host immune response, one for C. difficile activity, and another for microbiome community activity, each of which is a composite curve of the individual activities from each group, such as antibody production, neutrophil infiltration, toxin production, sporulation, and fiber and mucin degradation. Therefore, while we have identified populations of interest, it may be necessary to target multiple types of bacteria to reduce the community interactions contributing to host damage.

Here, we have shown several bacterial groups and their relative abundances are associated with variation in CDI disease severity. Further understanding how the microbiome affects severity in patients could reduce the amount of adverse CDI outcomes. When a patient is diagnosed with CDI, the gut community composition, in addition to the traditionally obtained clinical information, may improve our severity prediction and guide prophylactic treatment. Treatment of the microbiome at the time of diagnosis, in addition to C. difficile, may prevent the infection from becoming more severe.

## MATERIALS AND METHODS

### Animal care.

Six- to 13-week-old male and female germfree C57BL/6 were obtained from a single breeding colony in the University of Michigan Germ-free Mouse Core. Mice were grouped by bacterial community donor (M1, *n* = 3; M2, *n* = 3; M3, *n* = 3; M4, *n* = 3; M5, *n* = 7; M6, *n* = 3; N1, *n* = 11; N2, *n* = 7; N3, *n* = 3; N4, *n* = 3; N5, *n* = 3; N6, *n* = 3; N7, *n* = 7; N8, *n* = 3; and N9, *n* = 2), housed in cages at 2 to 4 mice per cage, and maintained in germfree isolators at the University of Michigan germfree facility. All mouse experiments were approved by the University Committee on Use and Care of Animals at the University of Michigan.

### C. difficile experiments.

Human fecal samples were obtained as part of a study by Schubert et al. and selected based on community clusters (18) to result in diverse community structures (see Table S1 in the supplemental material). Feces were homogenized by mixing 200 mg of sample with 5 mL of phosphate-buffered saline (PBS). Mice were inoculated with 100 μL of the fecal homogenate via oral gavage. Two weeks after the fecal community inoculation, mice were challenged with C. difficile. Stool samples from each mouse were collected 1 day prior to C. difficile challenge and plated for C. difficile enumeration to confirm no C. difficile was detected in stool prior to challenge. C. difficile clinical isolate 431 came from Carlson et al., had previously been isolated and characterized (35, 36), and was recently further characterized (37). Spore concentrations were determined both before and after challenge (79). A total of 10^3^ C. difficile spores were given to each mouse via oral gavage.

10.1128/mbio.01183-22.5 TABLE S1 Demographic information of subjects whose stool samples used to colonize germ-free mice. Download Table S1, PDF file, 0.02 MB. Copyright © 2022 Lesniak et al. 2022 Lesniak et al. https://creativecommons.org/licenses/by/4.0/ This content is distributed under the terms of the Creative Commons Attribution 4.0 International license.

### Sample collection.

Fecal samples were collected on the day of C. difficile challenge and the following 10 days. Each day, a fecal sample was collected and a portion was weighed for plating (approximately 30 mg), and the remaining sample was frozen at −20°C. Anaerobically, the weighed fecal samples were serially diluted in PBS, plated on TCCFA plates, and incubated at 37°C for 24 h. The plates were then counted for the number of CFU (80).

### DNA sequencing.

From the frozen fecal samples, total bacterial DNA was extracted using MoBio PowerSoil-htp 96-well soil DNA isolation kit. We amplified the 16S rRNA gene V4 region and sequenced the resulting amplicons using an Illumina MiSeq sequencer, as described previously (81).

### Sequence curation.

Sequences were processed with mothur (v.1.44.3), as previously described (81, 82). In short, we used a 3% dissimilarity cutoff to group sequences into operational taxonomic units (OTUs). We used a naive Bayesian classifier with the Ribosomal Database Project training set (version 18) to assign taxonomic classifications to each OTU (83). We sequenced a mock community of a known community composition and 16S rRNA gene sequences. We processed this mock community with our samples to calculate the error rate for our sequence curation, which was 0.19%.

### Toxin cytotoxicity assay.

To prepare a sample for the activity assay, fecal material was diluted 1:10 (wt/vol) using sterile PBS and then filter sterilized through a 0.22-μm-pore filter. Toxin activity was assessed using a Vero cell rounding-based cytotoxicity assay, as described previously (30). The cytotoxicity titer was determined for each sample as the last dilution that resulted in at least 80% cell rounding. Toxin titers are reported as the log_10_ value of the reciprocal of the cytotoxicity titer.

### Histopathology evaluation.

Mouse cecal tissue was placed in histopathology cassettes and fixed in 10% formalin, and then the samples were stored in 70% ethanol. McClinchey Histology Labs, Inc. (Stockbridge, MI), embedded the samples in paraffin, sectioned them, and created the hematoxylin- and eosin-stained slides. The slides were scored using previously described criteria by a board-certified veterinary pathologist who was blind to the experimental groups (30). Slides were scored as 0 to 4 for parameters of epithelial damage, tissue edema, and inflammation, and a summary score of 0 to 12 was generated by summing the three individual parameter scores. For nonmoribund mice, histopathological summary scores used for LEfSe and logistic regression were split into high and low groups based on greater or less than the median summary score of 5 because they had a bimodal distribution (*P* < 0.05).

### Statistical analysis and modeling.

To compare community structures, we calculated Yue and Clayton dissimilarity matrices (θ_YC_) in mothur (84). For this calculation, we averaged 1,000 subsamples or rarified samples to 2,107 sequence reads per sample to limit uneven sampling biases. We tested for differences in individual taxonomic groups that would explain the outcome differences with LEfSe (85) in mothur (using default parameters with an LDA of >4). We tested for differences in temporal trends through fitting a linear regression model to each OTU and tested for differences in regression coefficients by histopathological summary scores with LEfSe (85) in mothur (using default parameters with an LDA of >3). The remaining statistical analysis and data visualization were performed in R (v4.0.5) with the tidyverse package (v1.3.1). We tested for significant differences in β diversity (θ_YC_), histopathological scores, and toxin activity using the Wilcoxon rank sum test, nonunimodality to the nonmoribund histopathological summary score using Hartigans’ dip test, and toxin detection in mice using the Pearson’s chi-square test. We used Spearman’s correlation to identify which OTUs that had a correlation between their relative abundance and the histopathologic summary score. *P* values were then corrected for multiple comparisons with a Benjamini and Hochberg adjustment for a type I error rate of 0.05 (86). We built L2 logistic regression models using the mikropml package (87). Sequence counts were summed by taxonomic ranks from day 0 samples and normalized by centering to the feature mean and scaling by the standard deviation, and features positively or negatively correlated were collapsed into a single feature. For each L2 logistic regression model, we ran 100 random iterations using values of 1e0, 1e1, 1e2, 2e2, 3e2, 4e2, 5e2, 6e2, 7e2, 8e2, 9e2, 1e3, and 1e4 for the L2 regularization penalty, with a split of 80% of the data for training and 20% of the data for testing. Finally, we did not compare murine communities to donor community or clinical data because germfree mice colonized with nonmurine fecal communities have been shown to more closely resemble the murine communities than the donor species community (88). Furthermore, it is not our intention to make any inferences regarding human-associated bacteria and their relationship with human CDI outcome.

### Code availability.

Scripts necessary to reproduce our analysis and this article are available in the GitHub online repository (https://github.com/SchlossLab/Lesniak_Severity_mBio_2022).

### Accession number(s).

All 16S rRNA gene sequence data and associated metadata are available through the Sequence Read Archive via accession no. PRJNA787941.
