# Supplementary figures and images for: The Gut Bacterial Community Potentiates Clostridioides difficile Infection Severity
Source: mBio. 2022 Jul 20;13(4):e01183-22. doi: 10.1128/mbio.01183-22 (PMC9426473; doi:10.1128/mbio.01183-22)

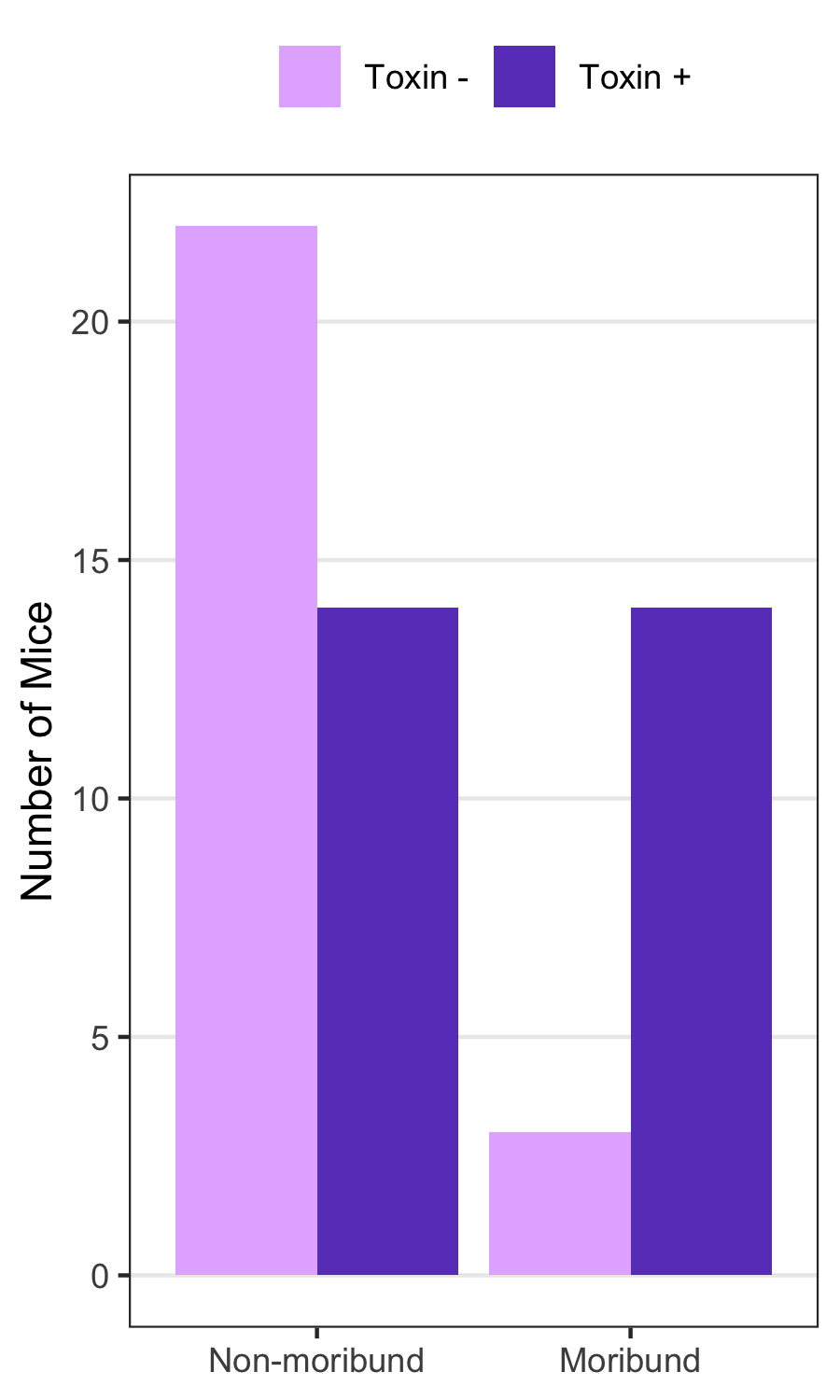

Supplement: FIG S1 [file mbio.01183-22-s0001.tif]

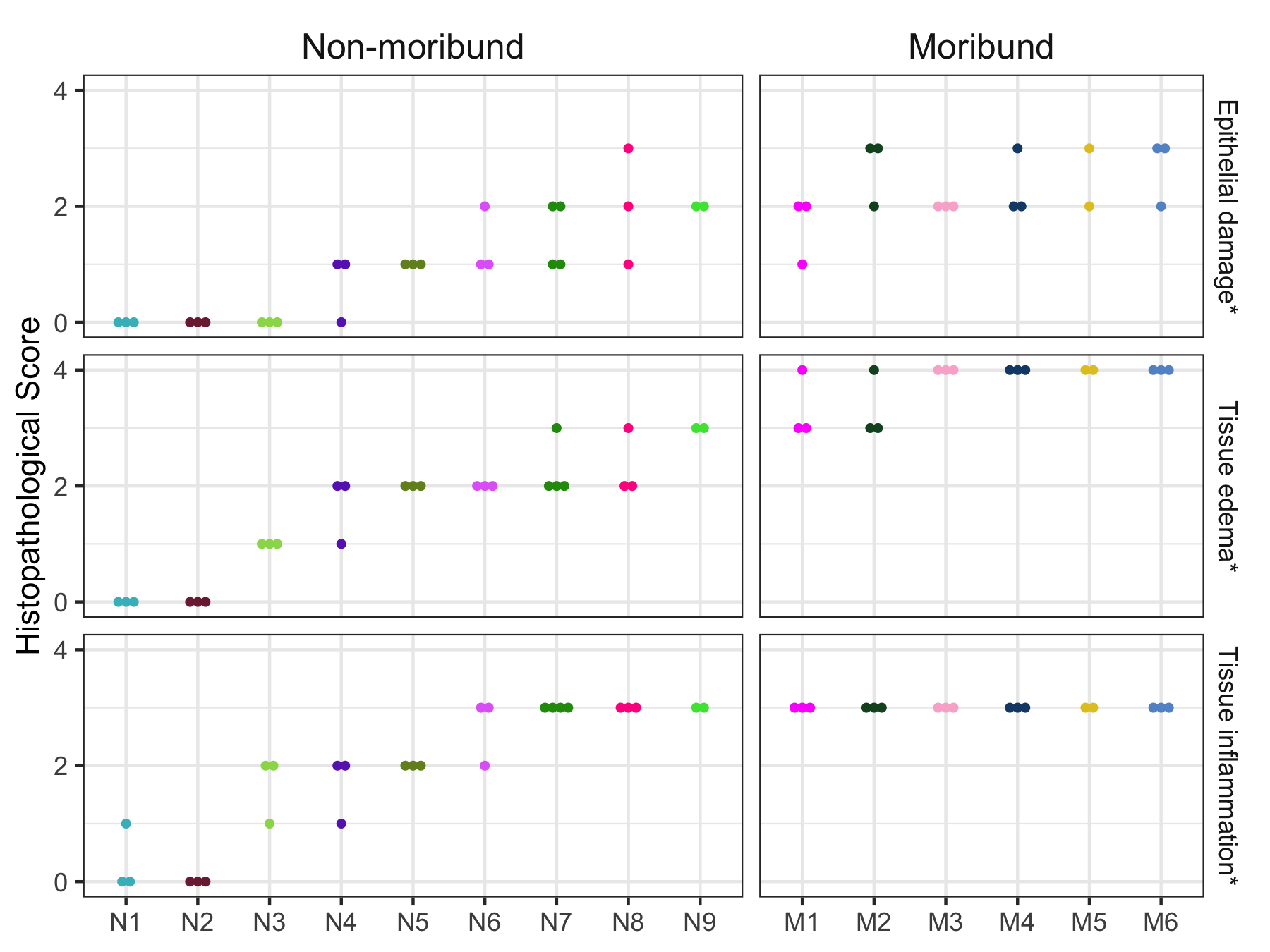

Supplement: FIG S2 [file mbio.01183-22-s0002.tif]

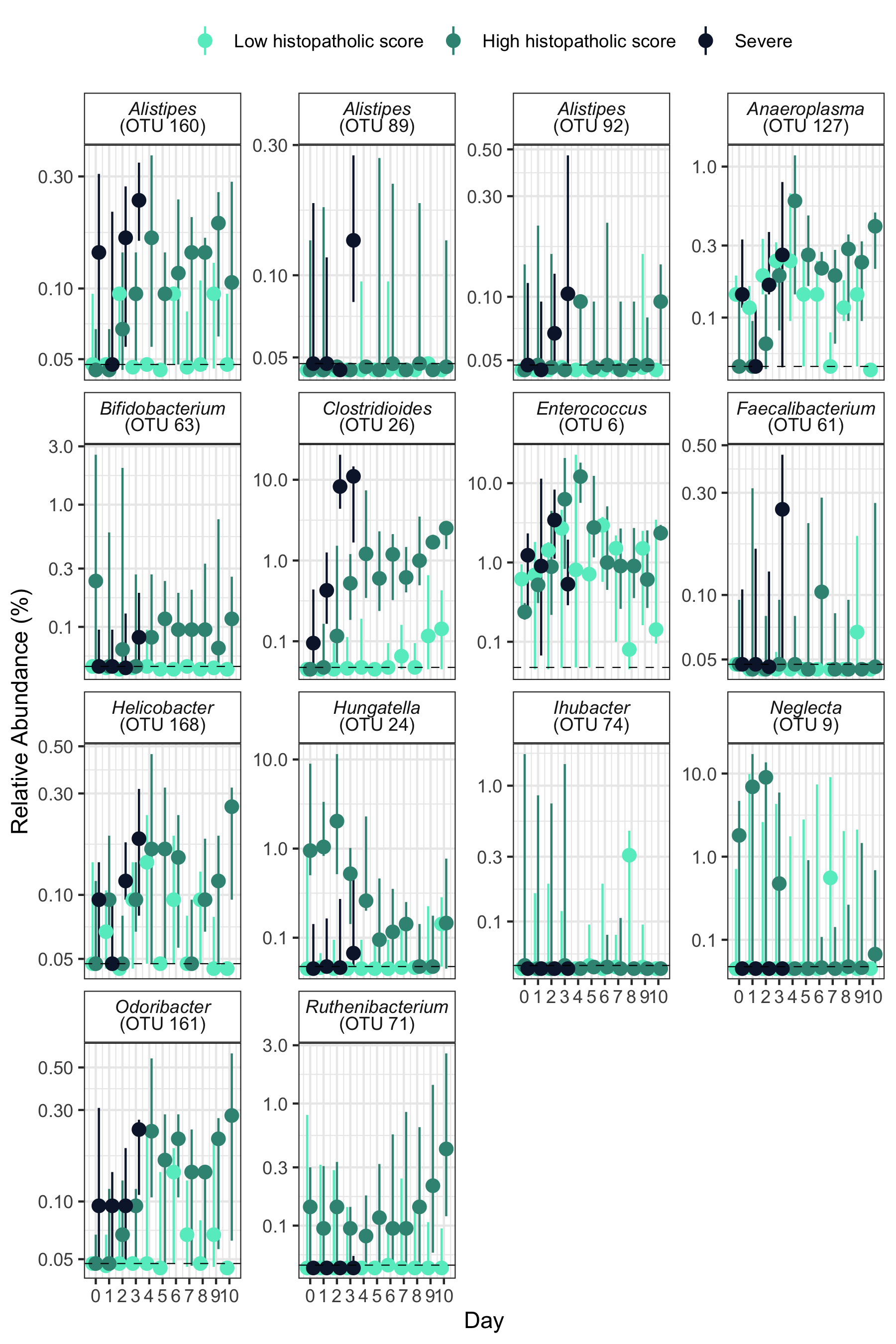

Supplement: FIG S4 [file mbio.01183-22-s0004.tif]

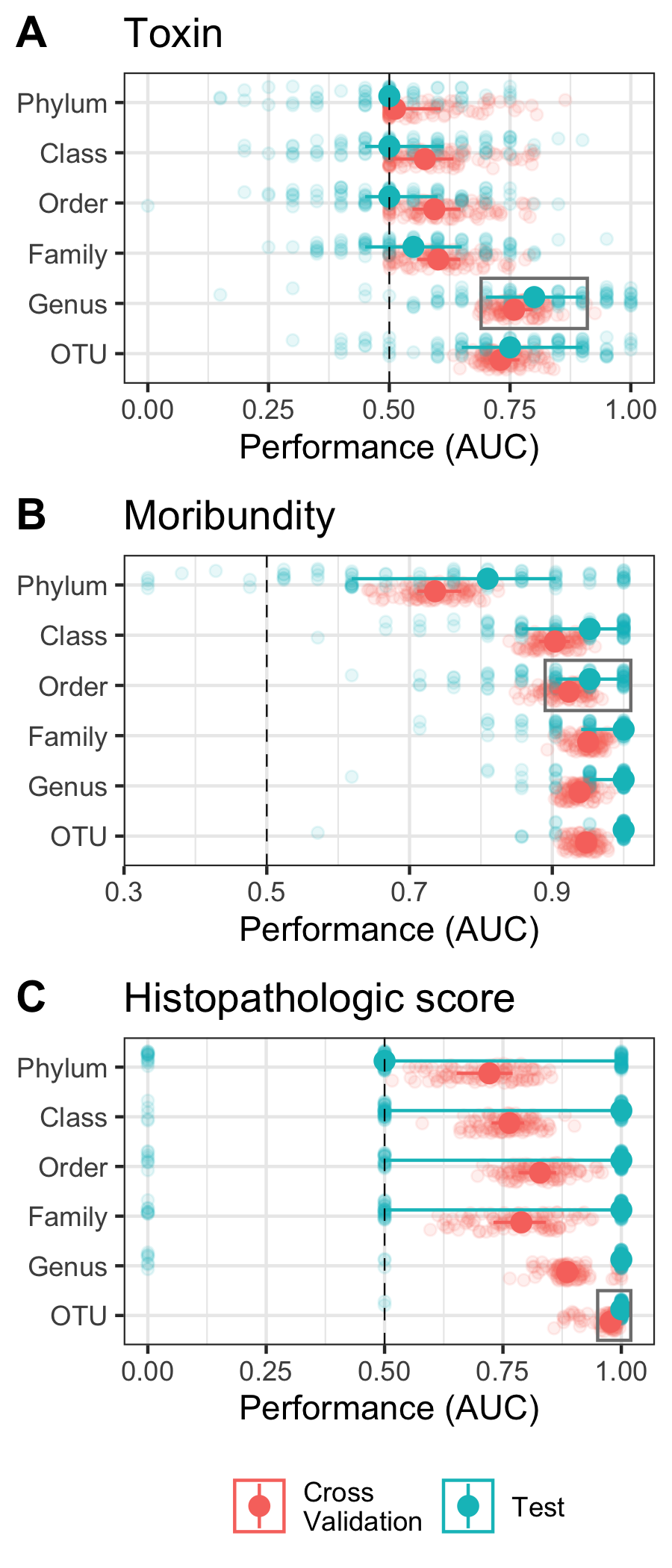

Supplement: FIG S3 [file mbio.01183-22-s0003.tif]
