# Supplementary material for: The Gut Bacterial Community Potentiates Clostridioides difficile Infection Severity
Source: mBio. 2022 Jul 20;13(4):e01183-22. doi: 10.1128/mbio.01183-22 (PMC9426473; doi:10.1128/mbio.01183-22)
Supplement: TABLE S1 [file mbio.01183-22-s0005.pdf]

## Table S1. Demographic information of subjects whose stool samples used to colonize germ-free mice.

| Sample Label | Subject ID | Subject Location | Sample Medium | Subject Age | Subject Sex | Recent Antibiotic Use | Proton Pump Inhibitor Use | H2 Receptor Blocker Use | Antacid Use | Healthcare Worker | History of CDI | Surgery within 6 Months | Vegetarian | Weight | Disease Status |
|---|---|---|---|---|---|---|---|---|---|---|---|---|---|---|---|
| M1 | DA10082 | community | anaerobic PBS | 72 | F | no | no | no | no | no | no | no | no | 200-249 | NonDiarrhealControl |
| M2 | DA00884 | hospital | carey-blair | 40 | F | no | no | no | no | yes | no | no | no | 100-149 | DiarrhealControl |
| M3 | DA10034 | community | anaerobic PBS | 21 | M | no | no | no | no | no | no | no | no | 150-199 | NonDiarrhealControl |
| M4 | DA01245 | hospital | carey-blair | 51 | M | yes | no | no | no | yes | no | no | yes | 200-249 | DiarrhealControl |
| M5 | DA00578 | hospital | carey-blair | 41 | F | yes | no | no | no | no | no | yes | no | 100-149 | DiarrhealControl |
| M6 | DA00431 | hospital | carey-blair | 55 | F | yes | yes | no | yes | yes | no | yes | no | Unknown | Case |
| N1 | DA00581 | hospital | carey-blair | 70 | F | yes | no | no | no | no | no | no | no | 100-149 | DiarrhealControl |
| N2 | DA00430 | hospital | carey-blair | 66 | F | no | no | no | no | no | no | no | no | Unknown | DiarrhealControl |
| N3 | DA01324 | hospital | carey-blair | 28 | F | yes | no | no | no | no | no | yes | no | 100-149 | DiarrhealControl |
| N4 | DA00953 | hospital | carey-blair | 77 | F | no | no | no | no | no | no | no | no | 150-199 | DiarrhealControl |
| N5 | DA10148 | community | anaerobic PBS | 25 | F | no | no | no | no | no | no | no | no | 100-149 | NonDiarrhealControl |
| N6 | DA01134 | hospital | carey-blair | 58 | M | yes | yes | no | yes | no | no | no | no | >=250 | DiarrhealControl |
| N7 | DA00369 | hospital | carey-blair | 78 | M | no | no | no | no | no | no | yes | no | Unknown | DiarrhealControl |
| N8 | DA10027 | community | anaerobic PBS | 52 | F | yes | no | no | no | yes | yes | yes | no | 150-199 | NonDiarrhealControl |
| N9 | DA10093 | community | anaerobic PBS | 27 | F | no | no | no | no | no | no | no | yes | 100-149 | NonDiarrhealControl |
